# Evolution of techniques for repair of intermediate-type bicuspid aortic valves

**DOI:** 10.1016/j.xjtc.2022.07.027

**Published:** 2022-08-24

**Authors:** Marc W. Gerdisch, Erle H. Austin, S. Russell Vester, George T. Stavridis, Arun Singhal, Alberto Weber, Marek A. Deja, Lawrence M. Wei, Vinay Badhwar, J. Scott Rankin

**Affiliations:** a Franciscan Health, Indianapolis, Ind; b University of Louisville, Louisville, Ky; c The Jewish Hospital, Cincinnati, Ohio; d Onassis Heart Center, Athens, Greece; e University of Iowa, Iowa City, Iowa; f Herzzentrum Hirslanden, Zurich, Switzerland; g Medical University of Silesia, Katowice, Poland; h West Virginia University, Morgantown, WVa

**Figure:**
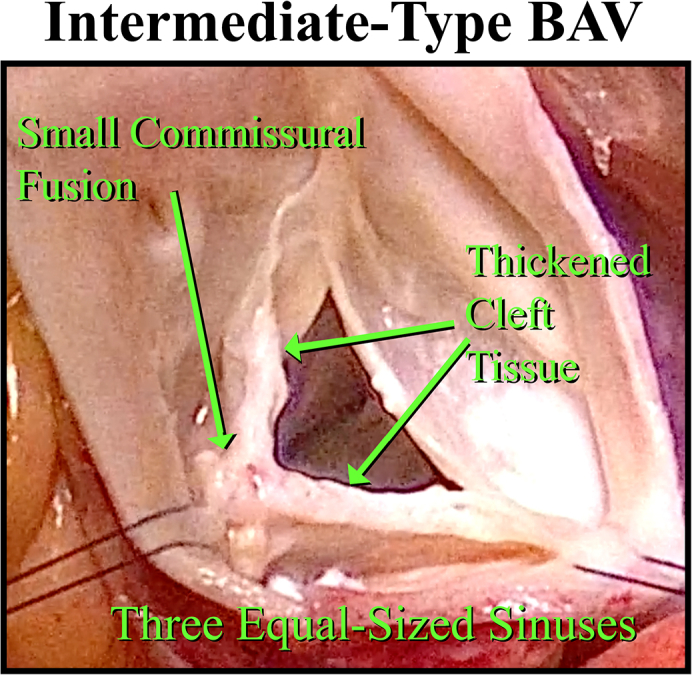
Features of intermediate-type bicuspid valves.

**Central Message**

Bicuspid valves with 3 equal sinuses have been difficult to repair. With geometric ring annuloplasty, repair with a 180° bicuspid ring has advantages of simplicity, reproducibility, and applicability.

With the goal of ensuring reproducibility for repairing all bicuspid morphologies, internal ring annuloplasty was developed both to correct annular dilation and to remodel each bicuspid aortic valve (BAV) to a standardized 180° 2-leaflet geometry—allowing surgeons to repair virtually all types of BAV defects.^1^^,^^2^ Implicit in aortic valve repair is the opportunity to minimize short- and long-term patient risk,^3^, ^4^, ^5^ which is especially appealing in BAV insufficiency, given the generally younger age of those affected. The development of techniques for managing leaflet geometry and associated degenerative changes have created this opportunity^2^^,^^6^ but also have defined the more challenging anatomies.^7^ Intermediate-type BAV (IBAV), sometimes referred to as form-fruste BAV,^8^ represents a spectrum of valve anatomy that is intermediate in sinus, leaflet, and commissural morphology between normal trileaflet and Sievers Type 1 bicuspid valves.^9^ In repairing IBAVs, surgeons must commit to either reconstructing the valve to a trileaflet anatomy or establish a well-functioning BAV. Superior event-free survival has been well documented for standard BAV repair,^10^, ^11^, ^12^, ^13^ but IBAV cases with 3 equal sinus segments have been more difficult.^7^ Application of routine repair to these more complex BAV anatomies would be a useful goal, and the development of aortic ring annuloplasty^1^ could help in standardizing surgical techniques. Our experience with IBAV repair encompasses many patients over the past 11 years; Video 1, Video 2, Video 3, Video 4 illustrate our technical evolution over this time in 6 representative patients.

## Methods

### Basic Anatomy of an IBAV Valve

**Figure 1:**
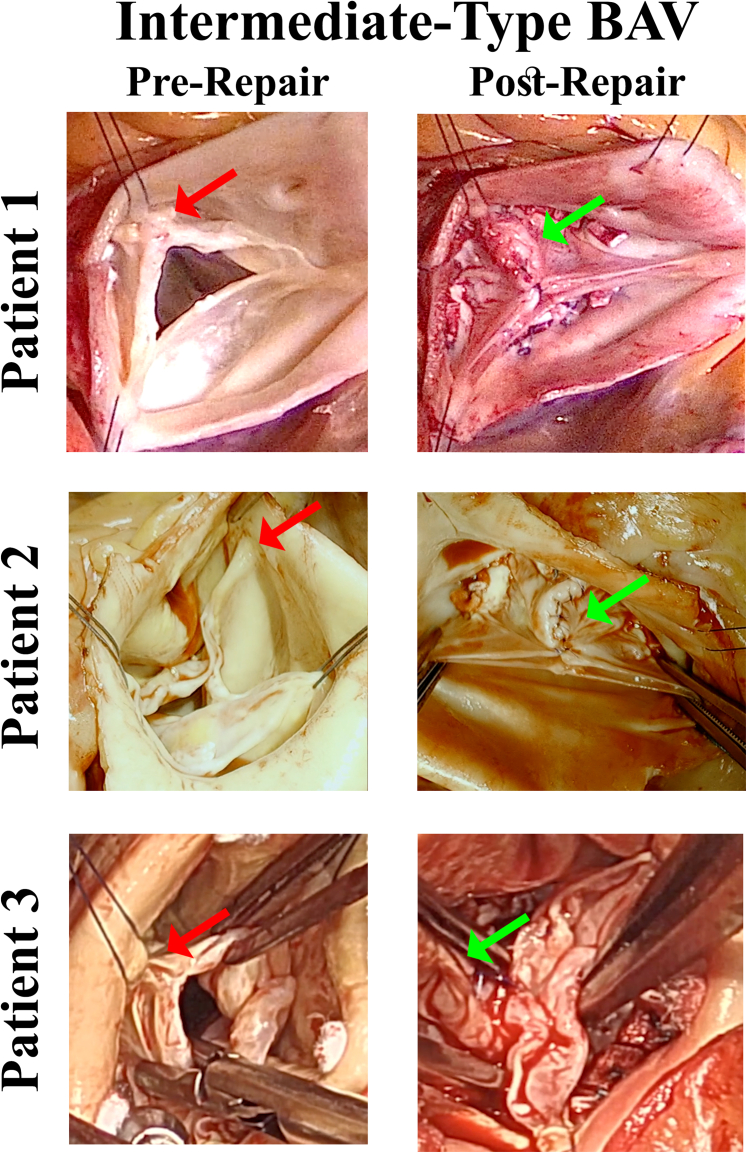
Anatomic features of the last 3 patients in the video presentations, repaired with 2-leaflet reconstructions. Patient numbers are the same as in Table 1 and text. Pre-repair, only small commissural fusions (*red arrows*), thickened cleft tissue, and 3 equal sized sinuses. Postrepair, the annuli have been reduced and remodeled into equal fused and nonfused segments by the annuloplasty ring; the clefts have been closed (*green arrows*); and both leaflets then coapted well with equal lengths and coaptation heights. *BAV*, Bicuspid aortic valve.

The anatomic characteristics of IBAV defects are intermediate between bicuspid and trileaflet geometry. Thus, a single commissural fusion exists (Sievers Type 1 anatomy), usually in the right–left commissure. However, the affected commissure has only a small fusion at the top of the commissure, and most of the commissure is a long cleft flanked by thickened cleft tissue with variable amounts of dysplasia (Figure 1). Frequently, the 3 sinuses are of relatively equal size, and the patients often are referred as trileaflet aortic insufficiency. Many have a posteriorly directed eccentric jet, similar to isolated right coronary leaflet prolapse,^14^ but in others, the jet is more central. In contrast to isolated right coronary leaflet prolapse, patients with IBAV usually are younger and do not have a leaflet fracture line and broken right leaflet tip. The posterior insufficiency jet is caused by malcoaptation of the cleft tissue and/or prolapse of the minimally fused right–left leaflet. Frequently, the leaflets are of 3 different sizes, with the noncoronary being slightly larger, and either the right or left being diminutive. Often, the right–left fused commissure is deficient and lower than the others. It is important to identify such valves as variant bicuspid defects because repair as a trileaflet valve often produces suboptimal results. The following 4 videos with 6 illustrative cases illustrate the technical evolution of our approach for IBAV repair—now to a high level of early and late success. Summary clinical characteristics of the 6 patients shown in the videos are listed in Table 1, with patient numbers consistent throughout.

**Table 1:** Summary clinical characteristics of the 6 patients shown in the videos∗

Patient	Age (y)	Prep NYHA class	Procedure	Preoperative AI grade	Postop AI grade	Postoperative mean gradient (mm Hg)	Follow-up (y)	Aortic clamp time (min)	CPB time (min)	Gender
1	60	3	AVr	4	0	8	6	163	196	M
2	9	2	Redo Ross AVr, ARA	4	0	16	2	178	232	M
3	53	2	AVr	4	0	13	2	135	166	M
4	40	3	AVr, AAA	4	0	11	2	213	252	M
5	72	3	AVr, AAA, CABG × 2, SA	4	0	8	0.5	277	351	M
6	47	2	AVr, AAA, ARA	4	0	4	0.5	133	145	M
Mean	46.8	2.5	–	4.0	0.0	10.0	2.2	183.2	223.7	100% M
SD	21.6	0.5	–	0.0	0.0	4.2	2.0	54.7	67.5	

*NYHA*, New York Heart Association; *AI*, aortic insufficiency; *CPB*, cardiopulmonary bypass; *AVr*, aortic valve repair; *M*, male; *ARA*, remodeling replacement of aortic root aneurysm; *AAA*, ascending aortic aneurysm replacement; *CABG*, coronary artery bypass grafting; *SA*, Cox atrial ablation.

∗ Ages ranged from 9 to 72 years, and all were male. All were experiencing heart failure from severe aortic insufficiency (AI), and all achieved Grade 0 AI after repair. Postrepair mean systolic gradients were low. Four of the 6 had additional aortic replacements and other procedures. Aortic clamp and bypass times are shown. Patient 4 required a permanent pacemaker implant at 6-months postoperatively. The repair in patient 1 seemed to be failing because of degeneration of inserted pericardial patch, but all others are doing well 1 to 3 years after surgery.

### Video 1 (Early Repairs Using Pericardial Commissural Augmentation)

In publications by Vohra and colleagues,^15^ IBAV was reconstructed by tricuspidization, which accommodated the presence of 3 relatively equal sinuses. Because the partially fused commissure was low, pericardial commissural augmentation was performed to raise the commissure and ensure good coaptation. This video illustrates a 35-year-old man (Patient 1) with heart failure, severe aortic insufficiency, and mild ventricular dysfunction, who was referred for repair of a trileaflet valve. Transesophageal echocardiography showed IBAV: the right–left fusion was minimal, the partially fused leaflet was prolapsing, and the 3 sinuses were equal in size. The eccentric insufficiency jet was posterior, the annulus was enlarged at 28 mm, and the left ventricle was dilating. On inspection, the right–left commissural fusion was small, and the commissure was open to the aorta. The leaflet free edge lengths sized to a 21 mm trileaflet annuloplasty ring, which was implanted under the annulus using standard techniques,^2^ except the elliptical trileaflet ring was turned 120° to shorten the distance from the noncoronary leaflet to the augmented commissure. Strips of glutaraldehyde-fixed autologous pericardium were sutured to the commissural aspects of the right and left coronary leaflets, up to the aorta above the low commissure. The noncoronary leaflet was raised with plication sutures. Postbypass echocardiograph showed a low gradient, good coaptation height, and negligible residual leak. At 3.5 years after surgery, mild-to-moderate valve insufficiency had reappeared, and the pericardium seemed to have calcified (Video 1). The patient remained asymptomatic. At 5.5 years, echocardiography showed only a mild leak, but, the mean gradient had increased to 21 mm Hg, suggesting degeneration of the pericardium with reoperation needed at some point. It is now understood that pericardial insertion during aortic valve repair introduces a high risk for degeneration,^16^ and IBAV reconstruction using pericardium has been abandoned.

### Video 2 (Trileaflet Repair of a Dilated Ross IBAV)

Patient 2 was a 9-year-old boy who underwent a Ross procedure 4 years earlier for BAV disease. He recently developed heart failure and worsening Grade 4 autograft insufficiency, associated with a 3.1 cm neoroot annular diameter. His autograft was believed to be trileaflet, but on closer inspection, a small fusion was present at the top of 1 commissure, consistent with an IBAV defect—but of the pulmonary autograft.^17^ The nonfused leaflet was 21 mm, but the other cusps were 19 mm. Because of nearly equal leaflet sizes, a trileaflet repair was chosen with a 19 mm trileaflet ring, again rotated 120°. After ring insertion, the leaflets were brought to the center of the valve, but all had different effective heights and free-edge lengths. Thickened cleft tissue was softened using the ultrasonic aspirator to allow better coaptation. Complex trileaflet plication was required, and at the end, all 3 commissural lengths were similar, with the leaflets coapting well in the midline. The supracoronary pulmonary autograft was replaced with a polyethylene terephthalate graft 7 mm larger than the ring, or 26 mm. After bypass, the leaflets moved well with trivial residual leak and a 16 mm Hg mean systolic valve gradient. Thus, IBAVs can be tricuspidized satisfactorily, but even with small disparities in leaflet size, complex plication can be required. A 2-leaflet repair probably would have been easier and now would be preferred in this anatomy because of simplicity and the ability to use 1-size-larger bicuspid ring.

### Video 3 (IBAV Repair as 3- and 2-Leaflet Reconstructions)

The first patient (Patient 3) was a 53-year-old man with heart failure, severe aortic insufficiency, and mild left ventricle dysfunction, referred for a trileaflet valve anatomy. Operative echocardiography showed a central aortic insufficiency jet with 3 equal sinuses and a small fusion at the top of the right–left commissure. On inspection, a small right–left commissural fusion was evident with commissural cleft thickening. The annulus sized to 25 mm, and the noncoronary leaflet was 21 mm, with smaller 19 mm right and left leaflets. A 19-mm trileaflet ring was implanted, turning the ring 120° to enhance coaptation to the noncoronary leaflet. After left leaflet plication, all cusps met well in the midline, with equal effective heights, good opening, trivial residual leak, and a 13 mm Hg mean gradient.

The second patient (Patient 4) was a 40-year-old man with a 5 cm ascending aortic aneurysm and severe aortic insufficiency, again referred as a trileaflet valve. Transesophageal echocardiography showed a severe central leak, normal aortic root size, and 3 equal sinuses—but suboptimal leaflet visualization. Intraoperatively, a small fusion at the top of the right–left commissure and thickened cleft tissue were evident. The annulus was 25 mm, the noncoronary cusp was 21 mm, and both right–left fused leaflets were 19 mm. This time, a 21 mm bicuspid ring was implanted with closure of the right–left cleft to coapt with the noncoronary cusp as a 2-leaflet bicuspid repair. Plication and cleft closure were performed until both free-edge lengths were 32 mm (half of a 21-ring circumference) with good coaptation heights. After bypass, leaflet motion was good with no residual leak and an 11 mm Hg mean systolic gradient. These 2 cases illustrate how either 3- or 2-leaflet repairs can be utilized for IBAV, if all leaflets are of adequate size. However, 2-leaflet repair with a 180° bicuspid ring is simpler, usually involves less difficult plication, and frequently allows 1-size-larger ring.

### Video 4 (Currently Recommended Technique of 2-Leaflet Repair for Most IBAVs)

Two further cases of IBAV repair (Patients 5 and 6) are presented to illustrate the current approach to reconstruction—routinely using a 2-leaflet repair and a bicuspid annuloplasty ring. The spectrum of IBAV is illustrated in these patients, from a very small fusion to 20% fusion of the commissure. This method is employed in most IBAV cases at present, independent of other variables, because it offers a high degree of simplicity, standardization, and reproducibility. Summary images of the pre- and postrepair valves of patients undergoing 2-leaflet reconstructions (Patients 4, 5, and 6) are shown in Figure 1. Table 1 illustrates clinical characteristics for all 6 patients with numbering consistent with the text patient numbers.

A waiver of informed consent was obtained from the Institutional Review Board of West Virginia University for retrospective analysis of de-identified clinical data (#2005016064; approval date May 29, 2020; Expiration date May 28, 2025). Additionally, another specific opinion supporting a waiver of informed consent was obtained from WCG Institutional Review Board (#1-1490881-1; approval date November 12, 2021).

## Discussion

In the original regulatory trials of geometric ring annuloplasty.^18^ several patients were referred for having trileaflet aortic insufficiency but were found to have IBAV anatomy intraoperatively. At the time, this defect was poorly understood, and after repair as trileaflet valves, the patients were left with chronic Grade 2 residual aortic insufficiency. Subsequently, the pericardial augmentation technique was described^15^ and implemented in our practice. However, recurrent aortic insufficiency was common due to degeneration of the pericardial augmentation patches (Video 1) (Patient 1). With more experience, strategies for complex leaflet plication were refined, and 3-leaflet repairs without pericardium (turning a trileaflet ring 120°) became more successful for anatomies with 3 adequate leaflets (Videos 2 and 3) (Patients 2 and 3, respectively). If either the right or left cusp was diminutive, a 2-leaflet repair was undertaken.^2^ Over time, it became evident that 2-lealfet reconstruction with a bicuspid ring was simpler in most cases, requiring less difficult plication and allowing placement of a larger ring (Video 3) (Patient 4). More recently, almost all IBAV cases have been repaired as 2-leaflet valves (Video 4) (Patients 5 and 6), and this approach currently is recommended.

Before geometric ring annuloplasty, repair of bicuspid valves with 3 equal sinuses was fraught with relatively higher repair failure rates.^7^ These also have been called very asymmetric bicuspid valves^8^^,^^19^ to differentiate them from Type 0 BAVs with 2 relatively symmetrical fused and nonfused sinuses. Although several surgical approaches have been used, such as reimplanting IBAVs into grafts with 180° commissural configurations^20^ and suture tailoring of the aortic root,^21^ it is often difficult to achieve fully symmetrical fused and nonfused sinus and leaflet geometry. In contrast, the BAV ring achieves symmetrical 180° fused and nonfused annular segments in all cases, no matter the baseline anatomy.^2^^,^^22^ In this way, problems associated with 3 equal sinuses in IBAV are overcome routinely, making simple 2-leaflet repairs highly reproducible. Finally, unicuspid valves frequently have 3 equal sinuses, and bicuspid ring annuloplasty also facilitates autologous reconstruction of unicuspid defects, without the need for pericardial augmentation.^23^

The timing of intervention for aortic valve insufficiency is tempered by several clinical and patient specific variables.^24^^,^^25^ In bicuspid disease, the decision to proceed with surgical therapy is influenced by ascending aortic pathology, symptoms, severity of valve leakage, and findings suggestive of left ventricle dysfunction. Further complicating decision making is the overall younger age of patients with BAV, for whom the consequences of aortic valve replacement are magnified. However, as evidence has grown from multiple observational studies, a trend has emerged toward earlier intervention with the recognition that symptoms may be absent or minimal while important negative milestones in functional parameters are passed.^26^ For mitral regurgitation, the evolution toward earlier treatment of asymptomatic severe disease has occurred, largely due to the superb results of mitral valve repair and the recognition that the heart could be spared the consequences of chronic volume overload. Similarly, the emerging excellent results observed after BAV repair^11^^,^^12^ could initiate a trend toward earlier surgical intervention for BAV insufficiency.

## Conclusions

IBAV defects can be mistaken as trileaflet valves and commonly have 3 relatively equal sinuses with only a modest right–left commissural fusion. The insufficiency jet can be more central through inadequately coapting cleft tissue. With deficiency of either fused cusp, 2-leaflet valve repair with bicuspid ring annuloplasty is readily undertaken. With better-developed leaflets, 3-leaflet repair can be performed, but often requires complex plication because of subtle differences in leaflet size. Even in these cases, our current approach has evolved to a 2-leaflet repair that offers a more predictable morphologic result and requires less complicated leaflet plication. Finally, major annular remodeling afforded by the bicuspid ring equalizes the fused and nonfused annuli and allows repair of any relative sinus configuration using standard leaflet reconstruction techniques. In the final analysis, the utility of this approach will depend on long-term follow-up studies of larger patient cohorts.

### Webcast

You can watch a Webcast of this AATS meeting presentation by going to: https://www.aats.org/resources/1491.

**Figure:**
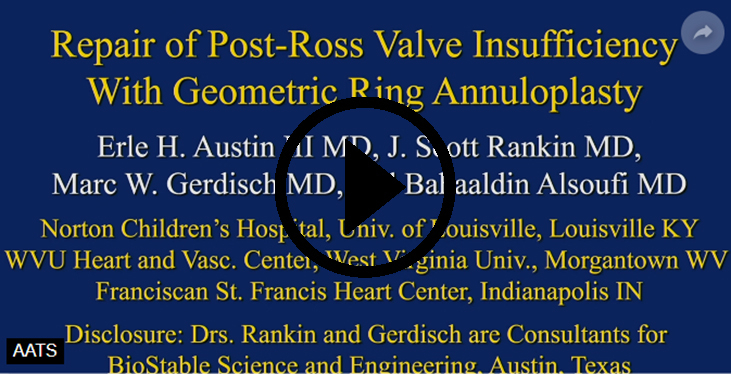

## Supplementary Data

Video 1 Repair of intermediate-type bicuspid aortic valve using a trileaflet annuloplasty ring and autologous pericardial commissural augmentation. Video available at: https://www.jtcvs.org/article/S2666-2507(22)00449-7/fulltext.

Video 2 Repair of an intermediate-type bicuspid aortic valve in a dilated Ross autograft using a trileaflet ring and complex commissural plication. This video illustrates difficulties sometimes encountered when plicating 3 different sized leaflets. Video available at: https://www.jtcvs.org/article/S2666-2507(22)00449-7/fulltext.

Video 3 Two patients are presented with intermediate-type bicuspid aortic valve, the first repaired to a trileaflet valve, and the second to a 2-leaflet valve. This video shows the ease of employing a 2-leaflet repair using a bicuspid ring. Video available at: https://www.jtcvs.org/article/S2666-2507(22)00449-7/fulltext.

Video 4 The final 2 patients are illustrated with intermediate-type bicuspid aortic valve repair using the recommended 2-leaflet reconstruction and bicuspid ring annuloplasty. Video available at: https://www.jtcvs.org/article/S2666-2507(22)00449-7/fulltext.
