# Supplementary figures and images for: Evolution of techniques for repair of intermediate-type bicuspid aortic valves
Source: JTCVS Tech. 2022 Aug 24;15:62–9. doi: 10.1016/j.xjtc.2022.07.027 (PMC9579988; doi:10.1016/j.xjtc.2022.07.027)

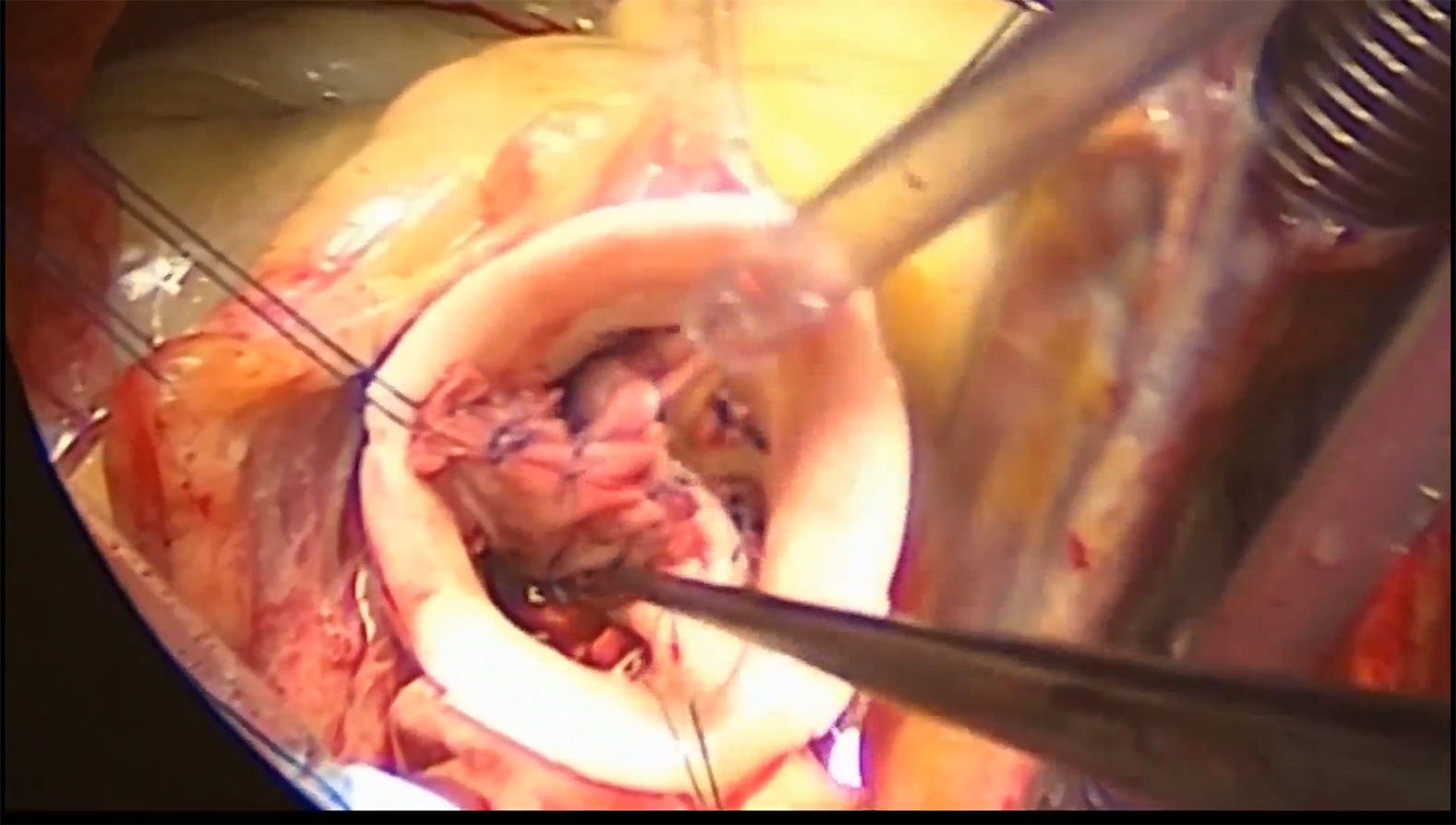

Supplement: Video 1 — Repair of intermediate-type bicuspid aortic valve using a trileaflet annuloplasty ring and autologous pericardial commissural augmentation. Video available at: https://www.jtcvs.org/article/S2666-2507(22)00449-7/fulltext. [file fx3.jpg]

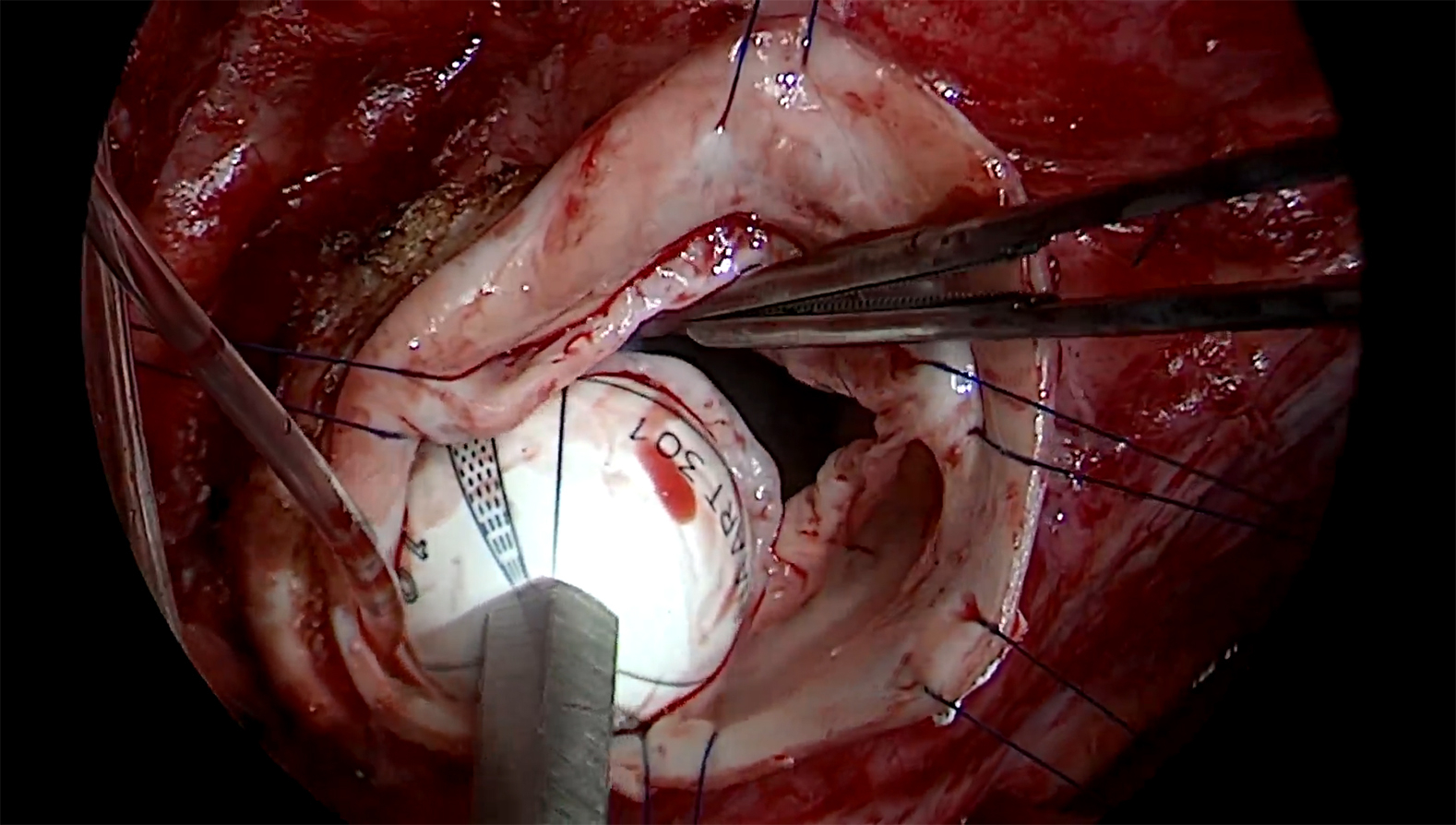

Supplement: Video 2 — Repair of an intermediate-type bicuspid aortic valve in a dilated Ross autograft using a trileaflet ring and complex commissural plication. This video illustrates difficulties sometimes encountered when plicating 3 different sized leaflets. Video available at: https://www.jtcvs.org/article/S2666-2507(22)00449-7/fulltext. [file fx4.jpg]

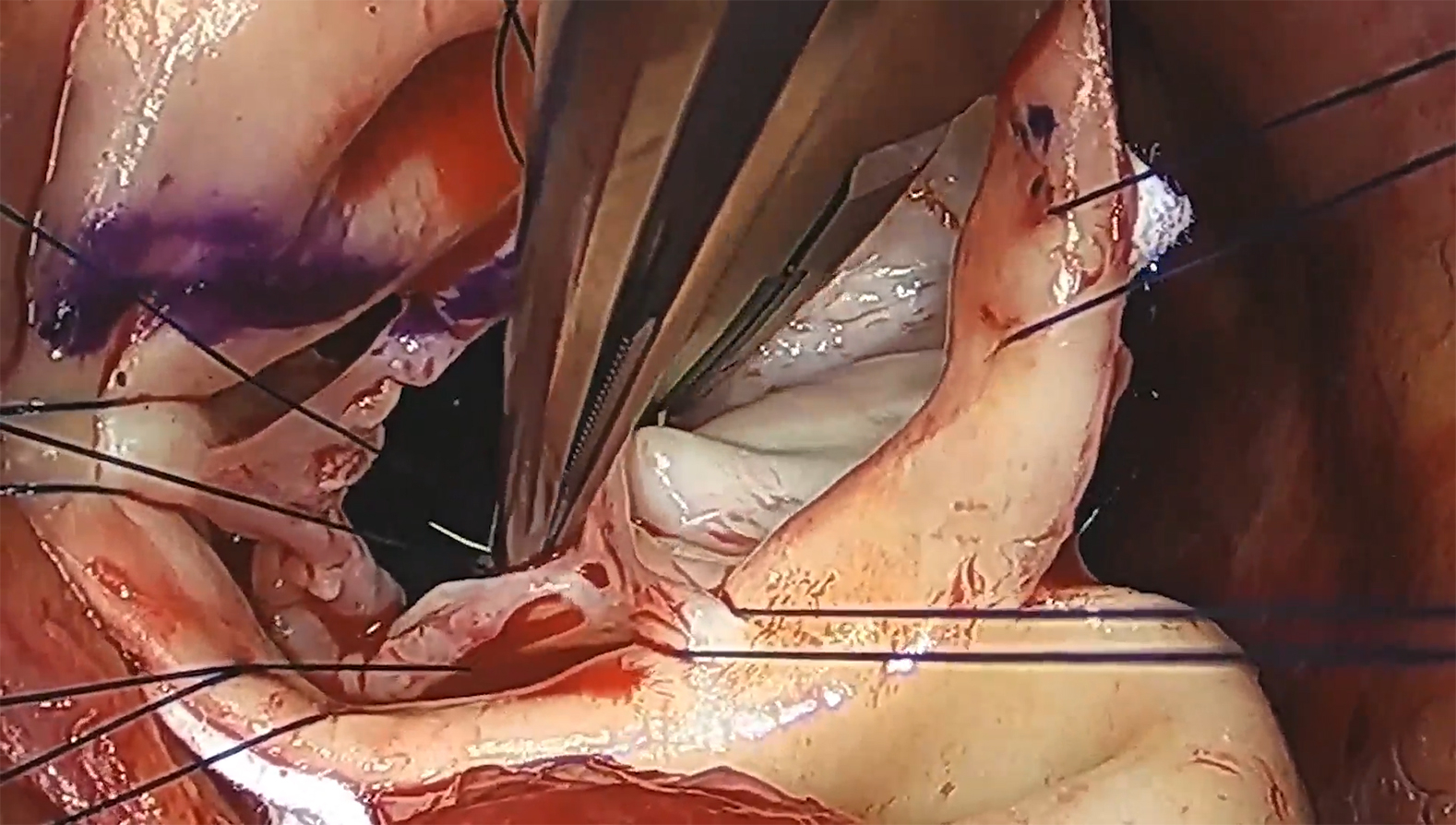

Supplement: Video 3 — Two patients are presented with intermediate-type bicuspid aortic valve, the first repaired to a trileaflet valve, and the second to a 2-leaflet valve. This video shows the ease of employing a 2-leaflet repair using a bicuspid ring. Video available at: https://www.jtcvs.org/article/S2666-2507(22)00449-7/fulltext. [file fx5.jpg]

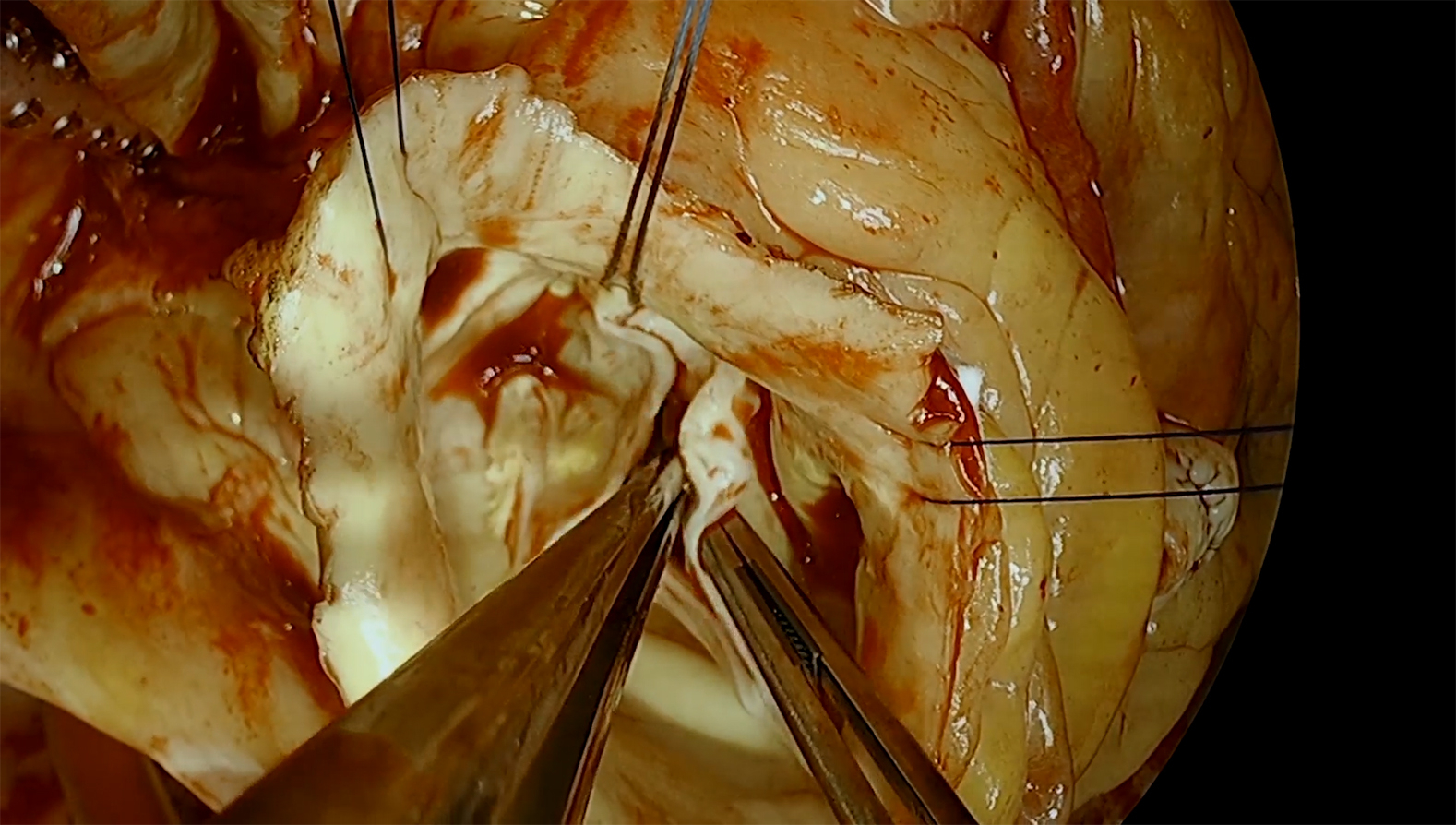

Supplement: Video 4 — The final 2 patients are illustrated with intermediate-type bicuspid aortic valve repair using the recommended 2-leaflet reconstruction and bicuspid ring annuloplasty. Video available at: https://www.jtcvs.org/article/S2666-2507(22)00449-7/fulltext. [file fx6.jpg]
